# Experimental approaches for manipulating choroid plexus epithelial cells

**DOI:** 10.1186/s12987-022-00330-2

**Published:** 2022-05-26

**Authors:** Ahram Jang, Maria K. Lehtinen

**Affiliations:** Department of Pathology, Boston Children’s Hospital, Boston, MA 02115 USA

**Keywords:** Choroid plexus (ChP), Cerebrospinal fluid (CSF), Blood-cerebrospinal fluid barrier (BCSFB), Gene therapy, Adeno-associated virus (AAV), Chemogenetics

## Abstract

Choroid plexus (ChP) epithelial cells are crucial for the function of the blood-cerebrospinal fluid barrier (BCSFB) in the developing and mature brain. The ChP is considered the primary source and regulator of CSF, secreting many important factors that nourish the brain. It also performs CSF clearance functions including removing Amyloid beta and potassium. As such, the ChP is a promising target for gene and drug therapy for neurodevelopmental and neurological disorders in the central nervous system (CNS). This review describes the current successful and emerging experimental approaches for targeting ChP epithelial cells. We highlight methodological strategies to specifically target these cells for gain or loss of function in vivo. We cover both genetic models and viral gene delivery systems. Additionally, several lines of reporters to access the ChP epithelia are reviewed. Finally, we discuss exciting new approaches, such as chemical activation and transplantation of engineered ChP epithelial cells. We elaborate on fundamental functions of the ChP in secretion and clearance and outline experimental approaches paving the way to clinical applications.

## Introduction

The choroid plexus (ChP) comprises a set of epithelial sheets sandwiching stromal cells and vasculature located in the lateral (LV), third (3V), and fourth (4V) ventricles of the brain. The ChP has several vital roles in the central nervous system (CNS). First, the ChP forms the blood-cerebrospinal fluid barrier (BCSFB), which is important for protecting the CNS from peripheral challenges including inflammation, pathogens, toxins [1–4]. Second, the ChP produces cerebrospinal fluid (CSF) into which it secretes nutrients and signaling molecules [5–10]. Third, it can perform CSF clearance functions including the removal of amyloid-beta [11] and ions (e.g. K^+^) under certain conditions [12]. In these capacities, the ChP-CSF system is an active player in brain development and lifelong brain health. ChP epithelial cell dysfunction is associated with several neurodegenerative conditions [1, 13–16]. Therefore, manipulating gene expression and functions of the ChP is appealing from basic science as well as therapeutic perspectives. As relatively long-lived cells in the CNS, ChP epithelial cells provide an attractive platform for gene therapy, scalable delivery of health-promoting factors for the brain, tissue engineering, and transplantation [5, 17, 18].

Many techniques have been applied to target ChP epithelial cells. For example, gene knockdown using small interfering RNAs (siRNA) can be used to silence gene expression in primary cultures of ChP epithelial cells and in vivo in mice [19–21]. In utero electroporation provides a powerful and rapid approach to deliver plasmid expression vectors for overexpression or knockdown studies in target cells and has been broadly used in the developing brain (e.g., cerebral cortex) [22]. Since the ChP is uniquely positioned in each brain ventricle and electroporation is directional [22], only a select population of ChP cells can be targeted by this approach at any time. However, electroporation of the embryonic 4V ChP has proven remarkably successful [19]. To effectively reach all ChP tissues in all ventricles, viral vectors and transgenic approaches have emerged as preferred strategies. Vectors including adeno-associated virus (AAV), adeno- and lentiviruses are commonly employed as carriers for gene delivery into the brain via intracerebroventricular (I.C.V.), intrathecal (I.T.), or intravenous (I.V.) routes. These techniques lend helpful temporal and spatial control to investigating ChP functions. Transgenic mice can be readily leveraged to delete or overexpress genes of interest in ChP epithelial cells.

Here, we review current experimental approaches available for targeting ChP epithelial cells, with a focus on in vivo strategies. We discuss the use of genetic reporter lines to visualize the targeted ChP, as well as the viruses used to target these cells for manipulation. We touch on the application of chemogenetic approaches using genetically engineered receptors to modulate activity of ChP epithelial cells. Finally, we provide a brief summary of ChP cell-targeted grafts and transplantation studies and their application to repair the damaged CNS.

### Genetic tools for targeting ChP

This section covers genetic approaches including traditional transgenic mouse models, inducible systems, cell-specific systems for loss or gain of gene function in ChP epithelial cells, and genome editing tools.

#### Reporter mouse lines

Reporter mice can facilitate tracing cell lineage and performing cell morphological analyses. They may also prove to be useful in the emerging ChP field of transplantation to evaluate cell integration and survival (see below). Reporter lines that enable tracking of labeled ChP epithelial cells have been established by leveraging the most highly expressed ChP signature gene, *Transthyretin (Ttr) *[23, 24]. TTR is secreted by ChP epithelial cells into the CSF, where it functions as a carrier protein for thyroid hormone (thyroxine_4_) [25]. An early *Ttr::RFP* reporter transgenic mouse was created with red fluorescent protein (mRFP1) [26]. Although broadly expressed across ChP epithelial cells during early embryonic development, *Ttr::RFP* transgene expression is lost in a fraction of ChP cells during later embryonic stages in this line. Labeling takes on a mosaic pattern in ChP, and expression is sparse postnatally [24, 27]. To achieve more complete postnatal expression, a human *TTR BAC (bacterical artificial chromosome)-tdTomato* mouse line was developed in which *Ttr* itself is replaced by *tdTomato*. In this line, robust *tdTomato* expression appears during early embryogenesis and remains stable well into adult life (e.g., up to 10 months) [27].

**Fig. 1:**
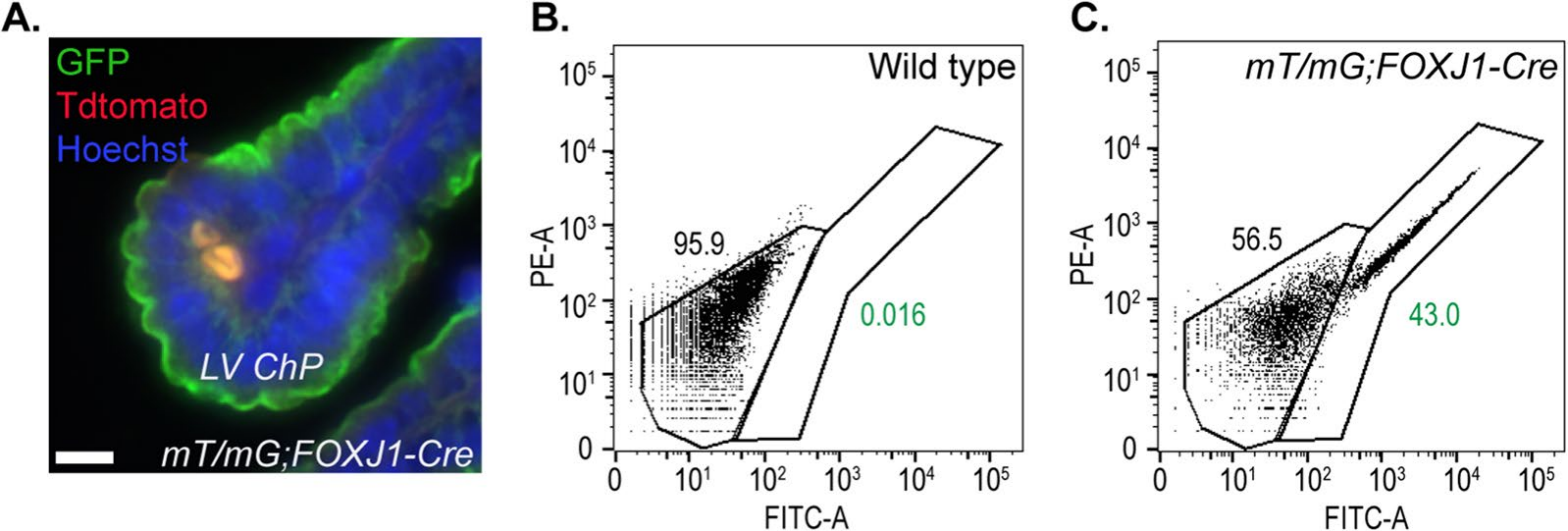
Representative GFP expression in LV ChP cells of wild type and *mT/mG;FOXJ1-Cre* mice. **A** *mT/mG;FOXJ1-Cre* reporter mice show GFP positive ChP epithelial cells immunostained with anti-GFP (green) antibodies and stained with Hoechst (blue) to mark nuclei. Scale bar, 10 μm [24]. Flow cytometry analyses of LV ChP cells of wild type (control, **B**) and *mT/mG;FOXJ1-Cre* adult mice **C** indicate that more than 40% of cells are mG-positive in *mT/mG;FOXJ1-Cre* adult mice. Experiment conducted as in [35], where recombination pattern of this reporter mouse was examined in lymphoid organs, thymus, and spleen

The transcription factor Forkhead box J1 (FoxJ1) can also be leveraged to target ChP cells. FoxJ1 is expressed by multi-ciliated cells throughout the body [28]. In the pre-natal mouse brain, ChP epithelial cells are the sole multi-ciliated cells contacting the ventricles. However, they lose this privileged status soon thereafter when multi-ciliated ependymal cells differentiate from radial glia and mature in the week following birth [29, 30]. Thus, depending on the experimental question, *FOXJ1-Cre* transgenic mice, in which the human *FOXJ1* promoter drives *Cre* recombinase expression, can be an excellent tool for studying ChP epithelial cells [28] (Table 1). When expressed, Cre catalyzes site specific recombination between two *loxP* (locus of crossing over in bacteriophage P1) sites [31–33]. Thus, Cre will excise DNA at two *loxP* sites, deleting the intervening DNA sequence [34]. *FOXJ1-Cre* mice can be crossed with a double-knock-in line in which all cells express a membrane bound form of *tdTomato* (mT) at baseline, and in the presence of Cre recombinase, the mT cassette is excised, enabling expression of the mG (GFP) cassette [35]. In this way, *mT/mG* mice crossed with *FOXJ1-Cre* transgenic mice reveal GFP-positive ChP epithelial cells (Fig. 1) [24].

**Table 1:** Overview of mouse Cre lines for targeting ChP

Mouse line	Age of Cre onset	Consequences	References
*FOXJ1-Cre*	Cre expression occurs in ciliated epithelial cells present in the ChP	Gene deletion in all ChP	[28, 49]
	Cre expression occurs in ciliated epithelial cells present in the ChP	Gene expression in all ChP	[12, 38]
*Nestin-Cre*	Around E10.5		[56]
*Foxj1-CreERT2::GFP*	Tamoxifen-inducible at E13.5 and confirmed at E15.5 in the reference		[66]
*Pax2-Cre*	Confirmed at E12.5 in the reference	Conditional gene deletion in 4 V ChP	[19]
*Wnt1-Cre*	Confirmed at E12.5 in the reference		[50, 52]
*Wnt1-Cre2*	Confirmed at E12.5 in the reference		[53]
*Foxj1-CreERT2::GFP*	Tamoxifen-inducible E10.5-E12.5 and confirmed at E12.5 in the reference		[53, 67]
*Gdf7-Cre*	Confirmed at E12 in the reference		[50, 68]
*Otx2-CreERT2*	Tamoxifen-inducible at E9 and, confirmed at E11 in the reference	Conditional gene deletion in all ChP	[68]
*LPV-Cre*.*0607*	Onset not specified, Adult expression in reference		[54]
TAT-Cre delivery	Adult (5 days after single injection)		[71–74]

The *FOXJ1-Cre* transgenic system has also been used to trigger gene expression in embryonic ChP epithelial cells to facilitate a variety of experimental paradigms. For example, EGFP tagged ribosomal subunit L10a can be expressed in ChP epithelial cells by crossing *FOXJ1-Cre* mice with *EGFP:L10a* BAC mice [12]; combined with tissue dissection, this approach enables translating ribosome affinity purification (TRAP) studies [36]. In another example, ChP epithelial cells can be targeted by crossing *FOXJ1-Cre* mice with *Ai95D* mice [37], a calcium reporter GCaMP6f line, to observe calcium activity in ChP epithelial cells [38].

#### Reporter zebrafish lines

Due to its large size and semi-transparent embryos, *Danio rerio* (zebrafish) provides another powerful vertebrate model for studying the brain’s ventricular system and ChP development [39]. Several brain pathologies associated with human diseases can also be modeled in zebrafish (e.g., autism spectrum disorder, depression, and Parkinson's disease) [40, 41]. A better understanding of ChP involvement may shed light on the molecular mechanisms of different neurological disorders and establish further tools for drug development [40–42]. In contrast to mammals, the zebrafish ChP is located close to the dorsal surface of the brain and is therefore experimentally more accessible. Thus, non-invasive in vivo live imaging is relatively straight-forward, making it possible to observe ChP development [43]. Early studies reported enhancer trap transgenic lines that express GFP in the zebrafish ChP [43–45], enabling the study of signaling molecules during development. One of these lines, the *Et(cp:EGFP)*^*sj2*^ line, which expresses GFP under the control of the epithelia-specific *keratin4* promoter, results in targeted GFP expression in diencephalic and myelencephalic ChP epithelial cells that correspond to 3V and 4V ChP in the mammalian brain, respectively. GFP expression in this line begins at the larval stage and is retained at least until 1 month of age [46]. Barrier proteins such as Claudins can also be harnessed to mark the ChP. While expressed more broadly, claudin5-GFP zebrafish provide another good marker of the ChP barrier [47]. Because the ChP has a very distinctive, fenestrated vasculature, using *Plvap*, a gene required for formation of fenestrated capillaries [(*Tg(plvap:EGFP) lines)*], or the *Tg(kdrl:EGFP)* endothelial cell-specific reporter line, have also proven effective for visualizing the tissue for imaging studies [48].

#### Genetic gain- and loss-of-function in mouse ChP

In studies using transgenic mice, various promoters can be used to disrupt gene expression in ChP epithelial cells, and the most common ones are summarized in Table 1. The *FOXJ1-Cre* line [28] represents one of the most widely used approaches to date. For example, the circadian rhythm of ChP epithelial cells was tested by deleting the essential circadian clock gene *Bmal1* in ChP (and all other multi-ciliated cells in the body) by crossing *FOXJ1-Cre* transgenic mice with floxed *Bmal1* mice (*Bmal1*^*fl*^*/*^*fl*^) [49]. Conditional knockout strategies can enable temporal and spatial control, which is necessary to determine the role(s) of specific target genes in certain locations during development. For example, *Wnt1-Cre* lines can be used to target 4V ChP where *Wnt1-Cre* deletes *Sonic hedgehog (Shh)* in the *Shh*^*fl/fl*^ line [50, 51]. However, due to findings that *Wnt1-Cre* transgenic mice show ectopic upregulation of *Wnt1* expression and unintended midbrain phenotypes, a newer, *Wnt1-Cre2* has been generated [52]. This new *Wnt1-Cre2* line has been used to delete *Meis1* in 4V ChP in a *Meis1*^*fl/fl*^ line [53]. Other cases of a 4V ChP conditional knockout of *Sox9* can be achieved with *Pax2-Cre* mice [19]. In addition, the lymphotropic papovavirus control region (LPVcr) allows generation of ChP conditional knockout by the Cre-recombinase/loxP system (e.g., *LPV-Cre.0607* transgenic line) [54].

Cre-based approaches can also be harnessed for gain-of-function studies and to model diseases of the CNS including ChP carcinoma [55–58]. While ChP epithelial cells differentiate from the roof plate and neuroectodermal lineage along the neural tube [59], they are not typically considered part of the *Nestin* lineage [60, 61]. Accordingly, *Nestin-Cre/Rosa*^*mTmG*^ reporter mice show limited recombination (GFP-positive cells) in ChP epithelium during embryonic brain development [56]. However, following birth, unexpected recombination occurs, first in ventral regions of the 4V ChP, followed by recombination throughout each ChP in each ventricle of the brain [56]. By the end of the first postnatal week, Cre recombination recognized by *Rosa*^*mTmG*^ occurs throughout each ChP in this line [56]. This postnatal ChP *Nestin-Cre* expression has been paired with *StopFLMYC* mice [62], in which a floxed stop cassette [*loxP-stop-loxP*] is located between a promoter and the gene of interest [63], the tumor oncogene *MYC*. Cre-mediated excision of the stop cassette drives human *c-MYC* overexpression in ChP epithelial cells, resulting in devastating ChP tumors, matching WHO Grade III carcinoma classification used in the clinical setting. These tumors form predominantly in the posterior domain of the LV ChP and 4V ChP [56]. ChP tumor models can also be generated when pairing *MYC* overexpression with *Trp53* deletion under the *Atoh1-Cre* line [57].

Tamoxifen inducible knock-in approaches can further refine temporal dynamics of Cre-based strategies. In this approach, Cre recombinase is fused to a mutant form of the estrogen receptor ligand binding domain (Cre-ER^T2^) and localizes in the cytoplasm [64]. Upon tamoxifen binding, Cre translocates to the nucleus where it catalyzes recombination [65]. In the ChP, the mouse *Foxj1* promoter-driven Cre^ERT2^ system (*Foxj1*^*CreERT2::GFP*^) [66] induced by tamoxifen at E10.5-E12.5 can be used for conditional ablation of gene expression during the early stages of ChP development, as was done with *Wnt5a* in 4V ChP epithelial cells [53, 67], resulting in altered morphogenesis of 4V ChP. To elucidate the critical role of transcription factor *Otx2* in the development and maintenance of the ChP, *Otx2-Cre*^*ERT2*^ mice were generated by replacing the genomic region spanning the *Otx2* coding sequence, and then crossed with *Otx2*^*fl/fl*^ mice [68, 69].

Another elegant loss-of-function technique entails I.C.V. delivery of TAT-Cre, a recombinant fusion protein of Cre and the cell-permeable TAT sequence, that is readily taken up by mammalian cells and catalyzes recombination [70]. When TAT-Cre is delivered into adult ventricles, it is taken up by ChP epithelial cells. In *Otx2*^*fl/fl*^ mice, TAT-Cre results in ChP-*Otx2* deletion, resulting in altered cortical plasticity and neurogenesis [71–73]. Similarly, TAT-Cre injection into adult *App*^*fl/fl*^ mice induces *App* knockdown in ChP, with downstream consequences on adult neurogenesis [74].

Tetracycline-controlled gene expression systems (e.g., Tet-ON) are commonly used to regulate gene expression and can be applied to the ChP [75]. In this approach, a gene of interest is placed under the regulatory control of the transcriptional activator rtTA (**r**everse **t**etracycline-controlled **t**rans**a**ctivator) and its **T**et **r**esponse **e**lement (TRE). Tetracyline (or a derivative such as Doxycycline, Dox) binding to rtTA induces a conformational change that allows rtTA to bind the TRE and induce expression of the target gene of interest [76]. To investigate Shh overexpression in ChP, transgenic lines overexpressing rtTA were generated under the *Ttr* and *Otx2* promoters (*pTtr-rtTA* and *pFuguOtx2-rtTA*) [75]. These mice were then crossed with *pTRE-mShh/d2EGFP* transgenic mice [77], in which Shh and GFP were expressed in the presence of Dox. While ChP-Shh expression is typically restricted to the 4V ChP in mice [23, 24], Dox administration to pregnant dams resulted in robust ChP-*Shh* mRNA expression in each ventricle’s ChP and expanded ChP growth [75]. ChP-Shh expression was also accompanied by *Gli1* and *Gli2* expression in cerebral cortical progenitors lining the CSF-filled ventricles, enlarged ventricles, and disrupted cerebral cortical development [75]. One limitation of the *pTtr-rtTA* line is that exhibits progressively diminished rtTA expression postnatally [75], similar to the *Ttr::RFP* line (see above [24]). However, *pFuguOtx2-rtTA* transgenic mice provide a suitable alternative with sustained rtTA expression postnatally, resulting in increased neural stem cell proliferation in the subventricular zone [75].

In addition to gain- and loss-of-function gene expression studies, mouse genetics approaches can be harnessed for ChP ablation. For example, *Gdf7* lineage cells are localized to the anterior domain of the LV ChP. Therefore, *Gdf7*-driven diphtheria toxin A chain expression can be used to ablate the anterior domain of the LV ChP [78]. Intriguingly, because the posterior domain of the LV ChP forms in a contiguous field with the anterior domain, the posterior domain also fails to form following anterior endotoxin expression [78]. Some mouse lines also may fail to develop a ChP, as is the case in the extra-toesJ (*XtJ*), which carries a *Gli3* deletion and fails to develop a LV ChP [79] as well as *Otx2*-deficient mice [68], where all ChP were affected when gene deletion was induced at E9.

#### New tools for genetic manipulation: CRISPR/Cas9

While most of the mouse lines discussed above were generated by traditional transgenic approaches, the advent of CRISPR/Cas9 methods has revolutionized mouse transgenesis [80, 81]. These newer strategies can relatively quickly introduce gene disruptions in mammalian cells. For example, use of the Easi-CRISPR (**E**fficient **a**dditions with **s**sDNA **i**nserts-CRISPR) targeting strategy enables progress from “concept” to “F1 founder mouse” in as little as three months [82]. It succeeds by leveraging injection of long single-stranded DNA donors with pre-assembled ribonucleoprotein complexes with two guide RNAs into mouse zygotes [82]. The CRISPR/Cas9 approach was used to study serotonin receptor signaling in ChP epithelial cells [38]. Antibodies for the 5-HT2C receptor are often unreliable, and hence, the addition of a fluorescent mRuby tag to the receptor in the *Htr2c*^*mRuby*^ line provides an opportunity to visualize the subcellular localization of this receptor on the apical and basal surfaces of ChP epithelial cells [38]. The ease of CRISPR/Cas9 approaches has the potential to generate large-scale genetics resources for the field. Applications now range from zebrafish, rats, and pigs to primates, opening avenues to unprecedented opportunities for CSF research [83, 84].

#### Limitations of mouse genetic model systems

Genetic model systems provide powerful and enduring tools for ChP targeting but are not flawless. One of the inherent challenges to using transgenic mice in the study of the ChP is that tissue and cell-type specificity are dependent on promoter specificity. While it has been desirable to find ChP-specific Cre drivers for selective targeting of ChP epithelial cells (e.g., *LPV-Cre.0607* [54]), single cell transcriptomics studies suggest no single gene is likely to be expressed only in ChP epithelial cells compared to all other cells throughout the body [23]. *Foxj1-Cre* transgenic lines are extensively employed for transgene expression in multi-ciliated cells including ChP epithelial cells. However, as mentioned above, the *FoxJ1* promoter also drives expression in other ciliated epithelial cells in the brain and body (e.g., ependymal cells) [28]. Thus, CreERT2-mediated Otx2 silencing occurs not only in the ChP but also in other parts of the brain [68]. Workarounds to achieve greater specificity include intersectional Cre approaches and direct I.C.V. delivery of Cre-expressing viruses such as AAV-Cre (see below). Limitations notwithstanding, genetic model systems provide highly valuable resources that enhance the study of the development of ChP epithelial cells and will continue to be optimized for investigating fundamental ChP biology.

### Viral approaches for targeting ChP

**Fig. 2:**
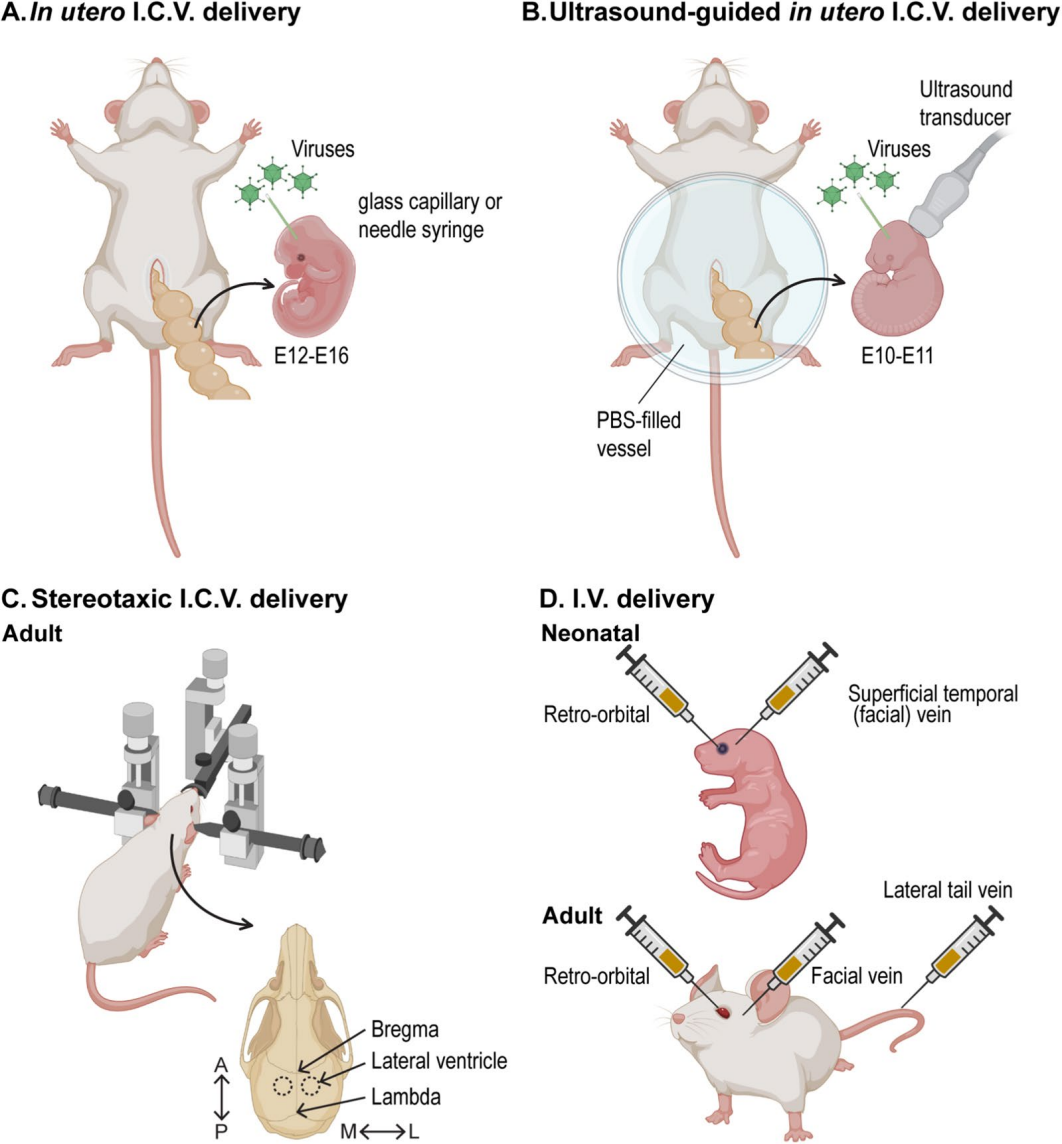
Commonly used viral delivery approaches for targeting mouse developing and adult ChP. Schematic of in utero I.C.V. **A** and ultrasound-guided in utero I.C.V. **B** delivery strategies for pre-natal mice. Schematic of adult stereotactic I.C.V. delivery **C** and various I.V. delivery routes in neonatal and adult **D** mice. Figure created with BioRender.com

The ability to manipulate genes with viral vectors has introduced a new flexibility for the study of the ChP. Long-term breeding can be avoided, and target gene expression in ChP epithelial cells can be obtained as soon as 24–48 h following vector introduction, making it possible to study acute effects of target gene manipulation in, for example, embryonic mice. Vector-based gene manipulation is particularly powerful when combined with transgenic technology as evidenced primarily by Cre-Lox experiments. Here, we review the DNA- or RNA-based viral vectors (e.g., AAV, adeno- and lentiviral vector systems) typically used for targeting ChP epithelial cells and then discuss the most used routes of viral administration at embryonic and adult ages (Fig. 2).

#### Adeno-associated virus (AAV)

**Fig. 3:**
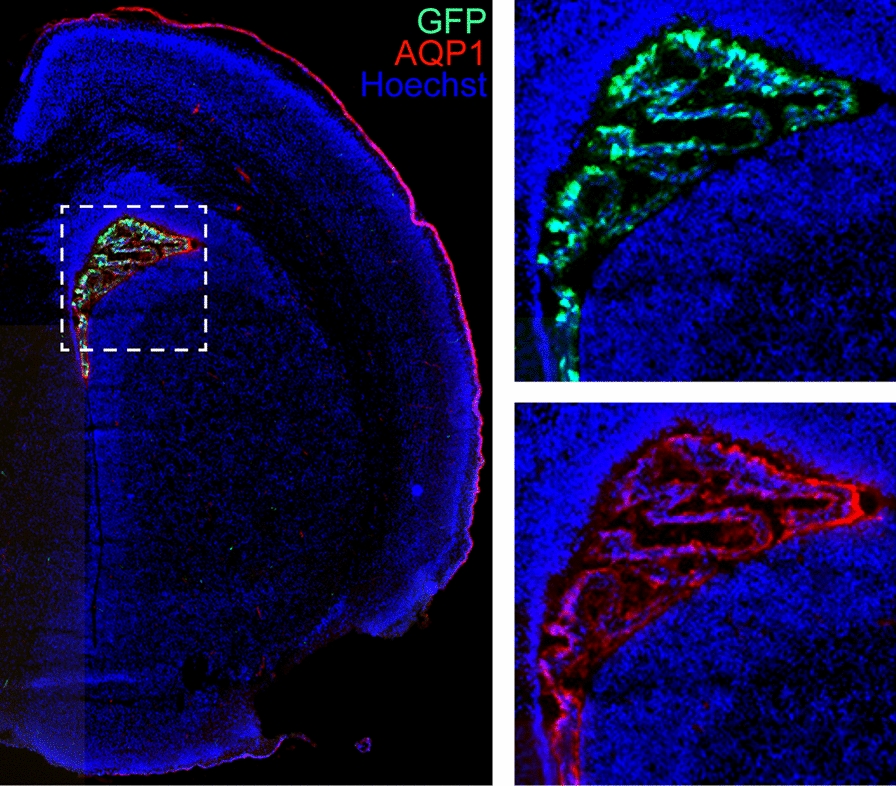
GFP expression in LV ChP following in utero I.C.V. delivery of AAV2/5-GFP. AAV2/5 delivered at E13.5 and immunostained 5 days later at E18.5 with anti-GFP (green) and anti-AQP1 (red) antibodies. Tissue stained with Hoechst (blue) to mark nuclei. Inset (dashed line box) highlights detection of AAV2/5-GFP and AQP1 expression in the ChP epithelium. Experiment conducted as in [53]

AAV vectors have cell type, tissue, and organ tropism depending on their serotype. In addition to tropism, viral titer, delivery route, animal genetics, and age also influence AAV gene expression. AAV2 is widely used to target the CNS but has not been noted to successfully transduce the ChP [85, 86]. By contrast, the hybrid serotype AAV2/5, which incorporates the genome of AAV2 and the capsid of AAV5, has proven tropism for ChP epithelial cells and produces robust expression (Table 2). Embryonic I.C.V. delivery of AAV2/5 is followed by ChP transduction in as soon as two days ([87] and Fig. 3), and gene expression can be sustained for months [88]. AAV2/5-mediated ChP gene expression in adult mice has been reported to occur within weeks of injection [89] and persists up to 12 months [74]. Other serotypes have been tested in several conditions with variable outcomes (see Table 2 and [89]); none have been as successful as AAV2/5. Importantly, genes introduced with AAV2/5 encode functional and demonstrably therapeutic proteins. For example, overexpression of the protein ATP7A with an AAV2/5 vector (AAV2/5-rsATP7A) mitigates the Menkes disease phenotype involving copper metabolism in the *Atp7a* knockout mouse model [90]. Overexpression of the Na^+^-K^+^-Cl^−^ co-transporter, NKCC1 [91], in developing ChP can modulate CSF-K^+^ levels and have long-term impact on ventricular size [12].

**Table 2:** Overview of AAV vectors delivered to mouse embryonic/neonatal/adult brains

Serotype/Capsid	Delivery route	Age at administration	Transduction^a^		Duration of transgene expression	References
			ChP	Ependyma		
AAV2/1	I.C.V.^c^	E15.5	Yes	Not tested	Up to 1 year	[152]
		P0.5^b^	Yes	Yes	1 month	[153]
		P0.5^b^	Yes	Yes	Up to 1 year	[85]
		8–12 weeks	Yes	Yes	1 year	[154]
		8–16 weeks	Yes	Yes	21 days	[89]
AAV2/2	I.C.V.^c^	P0.5^b^	Not tested	Yes (few)	1 month	[153]
		P0.5^b^	Yes (low)	No	Up to 1 year	[85]
AAV2/4	I.C.V.^c^	P0 or P1	No	Yes	4 weeks	[116]
		4–8 weeks	No	Yes	4 weeks	[116]
		6–8 weeks	Not tested	Yes	4 weeks	[155]
		85 days	Yes	Yes	Up to 160 days	[156]
AAV2/5	I.C.V.^c^	E10.5	Yes	Not tested	E16.5	[53]
		E13.5	Yes	No	E15.5	[87]
		E13.5	Yes	No	Up to E18.5	[53]
		E14.5	Yes	Not tested	P18-P24	[38]
		E15	Yes	No	130 days	[88]
		P0.5^b^	Yes	Yes	Up to 15 months	[92]
		P2-P3	Yes (specific)	No	Up to P300	[90]
		8–16 weeks	Yes (21 days)	Yes (6 months)	Up to 6 months	[89]
		3 months	Yes (specific)	No	Up to 12 months	[74]
	I.T.^d^	Adult	Yes	No	6 weeks	[93]
AAV2/8	I.C.V.^c^	8–16 weeks	Yes	No	21 days	[89]
AAV2/9	I.C.V.^c^	E15	Yes	No	130 days	[88]
		8–16 weeks	Yes (low)	No	21 days	[89]

^a^Expression beyond ChP and ependyma were reported. AAV2/5 shows the greatest tropism for ChP

^b^P (Postnatal day) 0.5: Day of birth

^c^I.C.V.: Intracerebroventricular delivery

^d^I.T.: Intrathecal delivery

As previously mentioned, mouse age and virus titer also influence AAV transduction. AAV2/5 delivered to neonates can transduce not only the ChP but also newborn ependymal cells lining the ventricles [92]. I.T. AAV2/5 delivery in adult mice leads to robust ChP transduction in each ventricle with the 4 V ChP having the highest expression, followed by the 3V ChP, and finally the LV ChP. This effect is likely related to a concentration gradient of virus exposure [93]. Consistent with this idea, the epithelial cells at the tips of the villi of the 3V and 4V ChP that are bathed by the CSF show higher rates of transduction, compared to epithelial cells at the base of the villi (see Supp Fig. 7D in [53]). While I.C.V. delivery of AAV2/5 shows tropism for ChP epithelial cells, I.T. injections reach several areas throughout the brain, with labeling including cerebellum, hippocampus, midbrain and olfactory bulb [93].

AAVs are widely used to deliver shRNA for knockdown studies or Cre recombinase to transgenic mice engineered with loxP sites for conditional gene deletion. For example, *Chd4* deletion in ChP epithelial cells can be achieved by in utero I.C.V. delivery of AAV2/5-Cre into *Chd4*^*fl/fl*^ mice [12]. Similarly, I.C.V. delivery of AAV2/5-Cre into adult *Klotho*^*fl/fl*^ mice deletes *Klotho* expression in ChP [94]. Remarkably, when delivered I.C.V., conditional gene deletion in ChP epithelial cells can be obtained without disrupting the same gene’s expression in nearby hippocampus [74]. AAV2/5-harboring *Otx2* shRNA injected I.C.V. into adult mice can also be used for *Otx2* knockdown in the ChP for at least three weeks following injection [73]. ChP-specific inhibition of miR-204 was achieved using AAV2/5 following adult mouse I.C.V. delivery [95].

AAV transduction can also be combined with CRISPR/Cas9 genome editing technology, as has been done with in utero I.C.V. injection of AAV2/9-PHP.eB-expressing gRNAs [96]. This approach achieves widespread, efficient gene targeting in the developing brain (e.g., Fig. 1C in [96]), which can be leveraged for gene deletion. Combining experimental strategies in this manner opens avenues for generating rapid knockout of ChP genes of interest and also helps address cell type specificity issues common to Cre-mediated approaches throughout the body.

Recent successes of AAV-based approaches that target neurons in brain regions affected by CNS diseases have ushered in an era of new hope that extends to the ChP and CSF [97]. Efforts are being taken to generate AAV variants with higher efficiency to cross the blood–brain barrier (BBB) and BCSFB so that they may reach the brain through the bloodstream. This strategy would also enable less invasive I.V. rather than I.C.V. injections for delivering genes to the ChP. The Cre recombination-based AAV targeted evolution (CREATE) strategy enables the development of AAV capsids that more efficiently transduce defined Cre-expressing cell populations in vivo [98–100]. For example, the AAV-PHP.B variant, generated using CREATE, transfers genes to target cells throughout the CNS with remarkably improved efficiency [98]. However, strain- and species-specific differences must always be considered. AAVs use multiple cellular receptors for attachment, internalization, and intracellular trafficking [101]. The AAV-PHP.B variant [98, 102] requires expression of the Ly6a receptor [102], revealing one host factor that underlies tissue tropism for this AAV variant. Because Ly6a is differentially expressed at the BBB of various mouse strains (e.g., present in C57BL/6 J but absent in BALB/cJ mice), its expression can pre-determine if peripherally injected AAV-PHP.B will cross the BBB. Accordingly, next-generation AAVs aim to demonstrate peripheral-to-CNS transfer and tropism to target cells of interest, so that systemic delivery routes can be used to lessen procedure invasiveness.

AAV vector safety and clinical efficacy have been demonstrated by promising Phase I/II/III clinical trials in various human disease settings, including lysosomal storage disorders (LSD) [103] and spinal muscular atrophy (SMA) [104–106]. Nonetheless, AAVs are known to trigger immune responses. Thus, off-target inflammatory effects should be considered in each experiment. Clinically, corticosteroids are often used to suppress unintended immune responses. In the case of AAVs, the genome of the vector can activate Toll-like receptor 9 (TLR9), which recognizes foreign DNA [107]. AAV vectors that are intrinsically less immunogenic have consequently been designed; these vectors incorporate short DNA oligonucleotides that antagonize TLR9 activation. This approach reduces innate immune and T-cell responses and has been tested in tissues including liver, muscle, and retina but may not be adequate for all immune responses of concern. A recent study also suggests that AAV transduction may be damaging to hippocampal stem cells residing in the dentate gyrus [108]. To our knowledge, hippocampal toxicity has not been reported with the AAV strategies used for targeting the ChP. However, all these points are important considerations for broadening therapeutic windows for AAV therapies and other DNA-based gene transfer methods.

#### Adenovirus

Similar to AAV vectors, adenoviral approaches confer notable advantages of long-term gene expression and reduced toxicity. However, adenoviruses tend to transduce a broader population of cells; for instance, adenoviral vectors delivered into adult rat ventricles revealed transduction in the ependymal cell layer and cervical spinal cord [109]. Moreover, while helper-dependent adenoviral (HDAd) vector injected into adult mice intrathecally exhibits ChP transduction as early as two days following injection, brain ependyma and other regions are also transduced and express the transgene [110].

#### Lentivirus

Lentiviral vectors offer an alternative and robust strategy for introducing proteins or peptides into the CSF via the ChP. One example comes from I.C.V. delivery of the vesicular stomatitis virus (VSV-G) vector into neonates, which results in transduction and long-term gene expression in ChP and ependymal cells [92]. Another valuable example comes from studies of Klotho, a transmembrane protein expressed by ChP epithelial cells and implicated in aging [111]. Sustained Klotho expression (for several months) was achieved with I.C.V. injected lentivirus in mouse models of Alzheimer's disease and cerebral ischemia [111–113]. In contrast to overexpression studies, lentiviral vectors can also produce loss-of-function by delivering shRNA. For example *klotho* shRNA-harboring lentivirus delivered I.C.V. into adult rats caused *klotho* knockdown in the ChP [111]. ChP-Megalin levels can also be modulated by lentiviral overexpression and RNA interference [21]. However, lentivirus ChP transduction is reported to be somewhat variable in adult mice [89].

To achieve greater cell-type specificity, two complementary lentiviral vectors can be used to achieve overexpression in ChP epithelial cells in a Tet-ON approach (similar to mouse genetic approach, see above). In this case, the *effector* virus contains a promoter that drives expression of rtTA and the GFP reporter [114]. The *target* construct includes the TRE DNA sequence, upstream of the gene of interest, followed by the RFP reporter. Expression of the *target* transgene is induced in the presence of Dox, which is known to cross the BBB (see Fig. 1 in [114]). Following I.C.V. delivery, replication-defective lentiviruses containing the *effector* and *target* integrate stably into the ChP epithelial cell genome for long-term expression. Mice provided with Dox in their drinking water then have expression of the target gene of interest. A more ChP-specific promoter for the brain (albeit with expression in other tissues throughout the body) was generated by isolating the 5’ flanking region of the corticotropin releasing factor receptor type 2 beta gene (CRFR2β) [114]. This lentiviral approach can result in expression of biologically active neuropeptides (e.g., corticotropin-releasing factor and gonadotropin-releasing factor) in the ChP, which are secreted into the CSF to great effect [114]. Gene expression can be induced as soon as two hours following Dox administration. The use of different ChP-brain-specific promoters may provide opportunities for fine-tuning transgene expression levels.

### Embryonic in utero I.C.V. injection

Various injection ages during development are reported, depending on the purpose of studies. In utero I.C.V. injection is widely used for delivering genes to target tissue. Following laparotomy, the virus is injected into the LV or 4V with a glass capillary tube or Hamilton syringe driven by a microinjector. The virus rapidly distributes throughout the ventricular system, transducing epithelial cells that contact CSF in each ventricle’s ChP. This approach is typically performed after the ChP has developed into a clearly visible anatomical structure (~ E12–E16) in order to provide sufficient numbers of epithelial cells for transduction [12, 53, 87, 88]. This time frame also corresponds to peak neurogenesis in the cerebral cortex [115]. However, tradeoffs need to be evaluated for each experimental age. At younger ages closer to E12, the injection procedure is easier owing to larger ventricles and limited surrounding brain tissue. In contrast, more ChP is available for transduction at E16, but the injection procedure becomes increasingly challenging due to the increased growth of the brain, skull, and more restricted access into the ventricles. Pairing I.C.V. injection with ultrasound imaging enables targeted viral delivery into even younger embryonic ventricles (E10-E11) to target ChP cells as they emerge. This age is more challenging to successfully target by eye due to the small size of the embryos and the opacity of the extraembryonic membranes, including the decidua. Ultrasound-guided viral delivery was recently optimized for E10.5 studies of early stages of ChP development [53].

### Adult I.C.V. delivery

I.C.V. or I.T. administration of gene vehicles can also effectively deliver viruses throughout the adult ventricular system. A stereotactic injection method is used for I.C.V. delivery for the adult ChP. Following head stabilization using a stereotactic frame, the injection site is determined by a designated 3-dimensional coordinate system of anterior–posterior (AP), medial–lateral (ML), and dorsal–ventral (DV) axes that aims for the lateral ventricle or cisterna magna. Example coordinates used for an adult (4–16 weeks) C57BL/6 mouse lateral ventricle are: 0.4 mm from bregma (AP), 1.0 mm (ML) and 2.0 mm (DV) from the brain surface [89, 116]. However, it is important to note that these coordinates may differ based on postnatal mouse age and strain. This approach has been successfully used in mice ranging from 8 to 16 weeks of age [74, 89], with enduring target gene expression up to and beyond 12 months [74].

Intraventricular delivery can be achieved by cisterna magna injection, lumbar puncture, or intrathecal catheter insertion. In mice, cisterna magna injection is the most commonly used intrathecal route. For example, helper-dependent adenoviral (HDAd) vector can be injected into the cisterna magna of 8–12-week-old C57BL/6 mice, and transgene expression can be observed in the ChP two days post-injection [110]. Alternatively, direct lumbar puncture of AAV into C57BL/6 mice shows clear transduction of each ChP throughout the ventricles along with various additional brain regions [93]. In general, the transduction pattern of CNS regions by I.T. injection reveals widespread and varying distribution that follows a caudal to rostral gradient of transduction.

The above procedures are all highly invasive. In an attempt to target other regions of the brain using less invasive approaches, both intranasal and I.V. approaches have been developed [97, 117–120]. However, these approaches require larger volumes of virus. New, more selective and potent viruses (such as the AAV-PHP.B as mentioned above) are being developed to circumvent these issues and will need to be tested on the ChP.

### Other approaches for targeting the ChP

#### Bioluminescence for studying the ChP in vivo

Understanding the functions of the ChP in vivo requires the ability to experimentally modulate its constituent cells, ideally by non-invasive means. Pharmacological and chemogenetic manipulations may provide innovative new strategies, taking advantage of unique direct chemical access to this brain structure through circulating blood and CSF [121].

The BioLuminescent-OptoGenetic (‘BL-OG’) [122, 123] method takes advantage of light that is produced by a chemical reaction within a cell when an enzyme (luciferase) oxidizes a small molecule (luciferin). While this approach is now established in neurons [122, 123], it has recently been adapted to the ChP [121]. In this approach, a bioluminescent reporter is expressed in ChP epithelial cells. A reporter molecule that expresses an optogenetic element is tethered to the bioluminescent luciferase enzyme. For example, the molecule LuMinOpsin3 (LMO3), a fusion of a bright and small luciferase enzyme (Gaussia; GLuc) and the light-activated *Volvox* channelrhodopsin (VChR1) [123–126], can be used. When GLuc binds its small molecule driver, the exogenously injected luciferin coelenterazine (CTZ), photons are released. The light is in turn absorbed by the VChR1, leading to a conformational change and allowing ions to diffuse down their concentration gradients. In neurons, this would lead to depolarization and firing of action potentials. Depending on the strength of the activation, the resting membrane potential of ChP epithelial cells can presumably be influenced transiently by this same approach, possibly with downstream effects on calcium-dependent cellular processes.

In ChP epithelial cells, luciferase expression can be driven by crossing a Cre line of choice (e.g., the *FOXJ1-Cre* mouse [28] with the *LSL-Lmo3-eYFP* [127] reporter mouse). These crosses strongly express LMO3 in the ChP, and bioluminescent signals can be imaged using a CCD camera via an implanted cannula. Luciferin cofactors can be administered by I.C.V., I.V., or intraperitoneal (I.P.) injection routes, providing readouts of the distinct pharmacokinetics associated with each delivery route. This approach therefore also sets the stage for future studies involving drug delivery.

The positioning of the ChP deep within the brain ventricles makes commonly used optogenetic methods with external light challenging [128]. The BL-OG approach provides an appealing workaround to that issue. It also provides optical confirmation, through bioluminescent light, of chemogenetic engagement on the target cell. In addition, the use of bioluminescence rather than fluorescence removes issues related to fluorophore bleaching and autofluorescence, thereby ensuring accuracy of estimates and providing a signal that is potentially detectable using non-invasive imaging methods.

### ChP tissue engineering and transplantation

Gene therapy paired with cell and tissue transplantation may provide a viable approach for future biological and clinical applications. Indeed, prior cell transplantation studies have suggested the therapeutic potential of ChP epithelial cells for brain injury and disease [129, 130]. For example, early studies hinted that transplanted ChP cells may have neuroprotective properties, especially in neurodegenerative disease models [131–133]. Human and mouse ChP epithelial cells can be derived from embryonic stem cells in response to bone morphogenetic protein 4 (BMP4), and these cells have self-assembling, secretory properties. Moreover, these cells can integrate into host mouse ChP epithelium [134], inviting the exciting possibility of harnessing this approach for intraventricular injections, transplants, and other interventions. In contrast to other body epithelia, mature ChP epithelial cells undergo surprisingly little proliferation or turnover under baseline conditions [135–137]. Recent attempts have succeeded in expanding cultured ChP epithelial cells in response to growth factors insulin-like growth factor-1 (IGF-1) and epidermal growth factor (EGF) [138].

Expanding on this foundation, a new methodology created 3-dimensional cultures of human ChP-like organoids [139] by treating human telencephalic organoids [140] with brief pulses of ChP-inducing signaling molecules (e.g., BMPs and WNTs) [134]. These predominantly epithelial cell organoids are well-developed and grow in vitro over extended periods of time, despite lacking vasculature and immune cells. Long-term culture of these organoids (e.g., 68–146 days) results in secretion of CSF-like fluid that approximates native human CSF. Because AAV-mediated approaches can be used for robust, long-term expression and secretion of factors of choice into the CSF [87], the future combination of viral and induced ChP tools can open doors to new therapies for a range of CNS disorders. Cultured ChP cells can also be transfected by polyamine-based transfection reagents to modulate gene expression [20, 141]. Collectively, these tools undoubtedly provide powerful platforms for drug screening. Leveraging these technologies for clinical use will require improved understanding of the survival of transplanted cells in vivo, endurance of viral transduction, and conflicting immune responses that may ensue.

## Conclusion

Here, we highlighted the current tools for manipulating the ChP, including transgenic mouse systems, viral vectors, pharmacological and chemogenetic manipulations, and ChP tissue engineering. These various approaches will contribute to understanding the function of ChP and further elucidate the ChP-CSF system. The spatial and temporal resolution offered by genetic approaches can better illuminate the many vital roles of ChP during development. Targeting the expression of critical genes in the ChP through gain- or loss-of-function studies will reveal their contributions to the growth and health of the CNS. In addition, targeting the ChP by specific AAV serotypes offers potential therapeutic strategies for neurodevelopmental and neurological disorders.

Even as current tools broaden our knowledge of in vivo ChP physiology, specificity remains a challenge. Combinatorial genetics and inducible Cre/LoxP systems and AAV vectors hold the most promise for enabling spatial and temporal control of gene manipulation. Inducible or cell-/tissue-specific promoters may also be applied to achieve specificity, and recent advances in optogenetics have welcomed a new era of photo-activatable Cre options [142, 143]. Although the I.C.V. administration route is well established and widely used to deliver viral vectors to the ChP mice of all ages, intranasal and I.V. routes are less invasive and technically easier—intranasal routes can bypass the BBB, and viral vectors that more selectively enter the brain are being developed (e.g., following I.V. delivery) [118, 144].

While we focus primarily on in vivo studies in this review, concerted efforts across many laboratories are aimed at improving in vitro approaches for investigating the ChP that should be amenable to the genetic targeting tools discussed in this review. These in vitro approaches include: primary ChP epithelial cells cultured in dishes and transwells [19, 145–147], cell lines such as immortalized mouse ChP epithelial cells (Z310 cells) [148] and porcine ChP epithelial cells (PCP-R) [149] and induced ChP cells [134]. Recently, ChP organoids [139] and ChP explants [38] were developed for investigating ChP functions and pre-clinical treatment strategies.

Recent advances that are beyond the scope of this review and were not covered include peptide- and ligand-mediated targeting of the ChP [150, 151]. Recombinant proenzymes and antibodies can be delivered by I.C.V. infusion in the clinical setting to target the ChP and treat neurologic diseases [150]. Early studies of ligand-mediated approaches also demonstrate that EGF-targeted phages can target the ChP epithelium and transduce genes ex vivo and in vivo [151]. Taken together with the aforementioned techniques, the next level of tools for targeting the ChP will have to be optimized properly; once they are, they will certainly contribute to a better understanding of genetic and molecular mechanisms of the ChP-CSF system and provide important insights to treat CNS disorders.

## Data Availability

Not applicable.

## Abbreviations

ChP: Choroid plexus
CSF: Cerebrospinal fluid
BBB: Blood–brain barrier
CNS: Central nervous system
BCSFB: Blood-cerebrospinal fluid barrier
AAV: Adeno-associated virus
I.C.V.: Intracerebroventricular
I.T.: Intrathecal
I.V.: Intravenous
Ttr: Transthyretin
LV: Lateral ventricle
3V: Third ventricle
4V: Fourth ventricle
gRNA: Guide RNA
CRISPR/Cas9: Clustered regulatory interspaced short palindromic repeats associated RNA-guided Cas9
LSD: Lysosomal storage disorders
SMA: Spinal muscular atrophy
